# Exploring small stories of older adults elicited by virtual nature videos with a randomized online survey

**DOI:** 10.1177/20552076241261886

**Published:** 2024-09-11

**Authors:** Kars Otten, Thomas JL van Rompay, Jan-Willem JR van ‘t Klooster, Debby L Gerritsen, Gerben J Westerhof

**Affiliations:** 1 Faculty of Behavioural, Management and Social Sciences, 3230 University of Twente, Enschede, The Netherlands; 2 RadboudUMC, Department of Primary and Community Care, Nijmegen, The Netherlands

**Keywords:** Older adults, social well-being, small stories, virtual nature, mystery, spaciousness

## Abstract

**Objective:**

Counteracting feelings of loneliness among older adults underscores the need to improve social well-being, for example, by sharing small stories. Interestingly, virtual representation of nature (VN) can stimulate social aspirations and trigger associations, which could be used as conversational material. Especially nature's characteristics of mystery and spaciousness seem promising. Therefore, it was investigated whether VN can elicit small stories in older adults using a randomized 2 (mystery: low vs. high) × 2 (spaciousness: low vs. high) design.

**Methods:**

In total 118 participants (60 years and older) were recruited. Small stories, nature-relatedness, available nature opportunities and demographics were measured. The small stories were analysed with respect to story elements (sum range: 0–4) and storytelling characteristics (ease of storytelling, valence, social intention).

**Results:**

The VN were able to elicited small stories: 97% (*N* = 115) contained at least one story element. Moreover, when participants felt more related to nature and assigned more positive valence to their story, they also had stronger intentions to use their story for social interaction. The VN characteristics of mystery and spaciousness showed no effects.

**Conclusion:**

Not so much the characteristics of nature (mystery and spaciousness) as the characteristics of the participants (nature-relatedness) played an important role in eliciting and sharing small stories.

## Introduction

The social well-being of older adults is a worldwide issue that needs to be addressed.^1^ Social well-being comprises several facets, including social integration and social identification, which refer to feelings of being socially connected to the community and others. Not feeling socially connected can be induced by factors associated with aging, such as a decline in health or physical disability, having dementia, retirement or losing a partner.^2^ Moreover, older adults reported that it is hard for them to cope with social isolation.^3^ This in turn can lead to more social isolation, e.g. by using passive coping strategies, requiring external support to break the pattern.^4^ This stresses the importance of interventions that aim to strengthen social connectedness in older adults.

A renowned means to promote social connectedness is storytelling,^5^ for example, by sharing ‘small stories’.^6^ Interestingly, experiencing nature can elicit a wide range of associations and thoughts useful for sharing small stories; e.g. memories.^7^ However, nature is not always available for people living in urbanized regions or for people with mobility constraints. Yet, this conflicts with the current importance and necessity of aging in place,^8^ which refers to being able to live relatively independently and to prevent or delay moving to a health care facility.^9^ In those cases, immersive technologies can help by bringing nature indoors and make its benefits available, although literature on using technology for aging in place is scarce.^10^ Therefore, this study investigated the potential of computer-animated nature videos as a means to elicit small stories, which in turn may improve the social well-being of older adults.

### Nature environments and social connectedness

In social connectedness interventions, the focus is on activities: e.g. therapy sessions and peer-discussion groups.^11^ But the role of the physical environment in which these social connectedness interventions occur is generally underacknowledged. Yet, reviews show that, in particular, exposure to nature environments, like forests or parks, has been shown to benefit social connectedness when compared with urban environments.^12,13^ For example, horticulture therapy, such as gardening, has social benefits for older adults in general and for people with dementia, in particular.^14,15^ Furthermore, persons with dementia and their caregivers acknowledge the importance of nature experiences for social aspects and reminiscing.^16^ Hence, exposure to real nature environments can be a means for promoting social connectedness among older adults.

For older adults, however, outdoor nature is not always available because they experience barriers related to health conditions and safety concerns when going out for a walk.^17,18^ Moreover, in highly urbanized environments one might not have the opportunity to experience outdoor nature because parks are too distant.^19^ So, especially when access to outdoor nature is limited, it seems relevant to bring nature experiences indoors for older adults to experience nature's beneficial effects.

To solve this issue, one can think of bringing real nature (e.g. plants) indoors using biophilic design strategies.^20^ Yet, such strategies may be difficult to implement because of hygienic, maintenance and financial considerations. Another solution could be using digital technology, as reviews point out that interventions using digital technology, e.g. enabling virtual social interactions, can have positive effects on several facets of social well-being, such as social support.^21,22^ Considering nature, indirect nature experiences can be created by using technology as well, e.g. watching nature videos or pictures.^23,24^ Particularly promising are newer technologies like virtual reality or augmented reality to make virtual representations of nature (VN). Although replacing outdoor nature experiences should not be the goal,^25^ VN can convey similar benefits as real nature environments.^23,26,27^ For example, the projection of animated nature landscapes resulted in engaging nature experiences and benefited social interaction among older adults^28^ and VN can positively affect prosocial aspirations and behaviour and increase feelings of connectedness to the community.^29,30^

When it comes to older adults, however, nature experiences seem to be overlooked when implementing technological solutions for their social well-being. Moreover, despite that a large body of literature shows positive effects of nearby outdoor nature on mental well-being, reviews and studies using VN are scarce and literature that did focus on VN or digital representations of nature, did not report on social connectedness.^31^ For example, in a previous study, older adults could cycle around a virtual lake using a chair-based bike in order to stimulate physical exercise.^32^ These findings highlight a research gap when it comes to studies that combine VN with social connectedness and storytelling, in particular. On a positive note, studies using VN agree on the importance of VN for people with limited access to outdoor nature.^31^ VN could therefore be a complementary and engaging means of nature interaction for people who have limited opportunities for outdoor nature experiences.

### Characteristics of nature environments

Despite the beneficial effect of VN on prosocial behaviour and social connectedness, literature is scarce concerning which nature characteristics are most beneficial for social well-being;^31,33^ e.g. tree density and the presence or absence of specific elements such as hills or a lake. Moreover, from a design and strategic point of view, evidence-based insights in what works and what does not are crucial for the selection and implementation of VN characteristics. Based on the Attention Restoration Theory (ART),^34^ and research on the emotion of awe,^35^ it was shown that especially mysterious and spaciousness nature environments can hold benefits for our well-being, as will be discussed next.

Mystery, i.e. unpredictability;^36^ is considered an important characteristic in ART as it stimulates curiosity and exploration by suggesting that there is more to experience if one would travel deeper into a nature scene.^34^ Examples include a winding path with no obvious destination or hills obscuring (parts of) the distant horizon. Nature images that are more mysterious resulted in better cognitive performance,^37^ creative performance,^36^ visual engagement^38^ and more personal engagement and more positive associations.^39^ Creativity, engagement and (positive) associations could be important to trigger storytelling, as it might be easier to come up with a small story when VN is engaging and stirs the imagination. It is therefore expected that more mysterious nature scenes have a positive effect on storytelling.

Spaciousness, i.e. extent;^34^ is an important nature characteristic in ART as it is associated with overview and opportunities for exploration. Moreover, spacious nature environments are linked to the emotion of awe, which in turn is related to a sense of connectedness to others and the world at large.^12^ In nature environments, such as parks and forest, spaciousness can be related to tree density, with more spacious environments having a lower tree density.^29^ Relevant for storytelling, more spacious nature environments can enhance social aspirations and parks with a spacious layout stimulate personal reflection when visitors are invited to write down their thoughts.^29,40^ Contrary, less spacious nature images trigger more associations: e.g. social contact, reminiscing or relaxation^39^ and evoke more visual engagement.^38^ The latter findings could mean that there is literally ‘more to see’ in less spacious nature environments. Moreover, when personal safety plays an important role, more intimate (less spacious) nature settings might be preferred as they are more readily associated with security and interpersonal closeness.^41^ In light of these findings underscoring the importance of spaciousness for pro-social behaviour and social well-being, spaciousness was manipulated in the current study to explore how spaciousness impacts storytelling.

In addition to nature characteristics, the effects of nature environments vary with an individual's physical and mental relatedness with nature.^33^ This nature-relatedness can influence people's interaction with nature, for example, people with higher nature-relatedness spend more time in nature and are more concerned about how their behaviours affect nature.^42^ Moreover, having more nature opportunities available nearby has a positive effect on perceived social support^43^ and mental health.^19^ Finally, older adults with higher nature-relatedness and more available nature opportunities have more associations with VN.^39^ Consequently, the effect of nature-based storytelling on social well-being is expected to be more pronounced for older adults with higher nature-relatedness.

### Storytelling

With storytelling, a storyteller incorporates parts of their identity into the story; i.e. stories reveal to others what kind of person the storyteller is.^5^ This personal information can be used to connect with others, since storytelling is shown to promote empathy, encourage the development of new social bonds and strengthen intimacy and bonding in existing relationships.^44,45^ Therefore, interventions aiming to promote storytelling are suitable methods for encouraging social connectedness.

Traditionally, storytelling refers to ‘big stories’ which encompass an autobiographical narrative obtained via several sessions of interviews.^46^ Such a thorough process leads to structured and understandable stories containing all four basic elements of narrative; i.e. ‘story elements’: situatedness, event sequencing, worldmaking/disruption and what it's like [to experience the events in the story].^47^ ‘Situatedness’ refers to stories occurring in a certain context and ‘event sequencing’ refers to how a story links events occurring across time. The element of ‘worldmaking/disruption’ implies that a story is used by the storyteller to make sense of the world. ‘What it's like’ refers to descriptions of how it feels like for the storyteller to experience the events mentioned in the story. Therefore, in the more traditional line of argument, if stories contain more story elements they are more in line with a typical story structure, which possibly makes them more likely to engage others and to increase social connectedness.

In everyday social interactions, however, stories might not contain every element and can then be referred to as ‘small stories’.^6^ Since direct exposure to nature environments might not immediately lead to a clearly structured story, the concept of ‘small stories’ seems applicable for investigating storytelling elicited by nature environments. Support for this claim comes from studies showing that both real and virtual nature experiences trigger precursors of small stories: positive and personally engaging associations^39^ and positive and exciting thoughts, positive feelings and memories.^7,48^ In this line of argument, even if not all story elements are incorporated in a story, it might still be worthwhile and meaningful to others and subsequently, useful to increase social connectedness.

Moreover, the current study recognizes that for storytelling to promote social connectedness the story should be worthwhile to tell others.^49^ To capture this tellability, three ‘storytelling characteristics’ were of interest: ease of storytelling, valence and social intention. A small story that is easier to produce might also be more worthwhile to tell others. Moreover, positive valence and social intention are of interest, because positively valenced memories can be used to experience enjoyable social interactions.^50^ Social intention was considered the most important storytelling characteristic as this is the most direct determinant of actual storytelling.

Recently, reviews show that technology has been integrated successfully in storytelling interventions, so-called: digital storytelling.^51,52^ Importantly, benefits on social engagement and quality of relationships have been found in people with dementia. Nevertheless, with the present importance of aging in place and the benefits technology can bring,^10^ digital storytelling interventions might also be useful for older adults who do not have dementia but do experience feelings of loneliness and could benefit from such interventions that improve social well-being.

Almost all reviewed studies on digital storytelling take a ‘big story’ approach. In most, stories were obtained via several (semi-)structured storytelling sessions over a period between six to 52 weeks. Although literature is more scarce, one study did show that a technology-based small story approach might be effective to improve social well-being.^53^ Yet, the studies discussed do not consider effects of nature environments. This again highlights the research gap when it comes to studies that combine VN with storytelling. The limited research nearest to this topic suggests that VN might prompt storytelling in the form of ‘small stories’ because it can elicit positive associations, personal engagement and memories.^7,39^ Moreover, nature's characteristics of mystery and spaciousness might be the most effective for this purpose. As such, the question remains if and how VN can elicit small stories.

Therefore, this study will address the research question to what extent VN are able to elicit small stories using the presence of ‘story elements’ and ‘storytelling characteristics’ in participants’ responses (RQ1). In addition, it is expected that higher levels of nature-relatedness positively influence the number of story elements and the storytelling characteristics (H1); VN high in mystery (compared to low) elicit small stories with more story elements and are positively related to storytelling characteristics (H2); and spaciousness will have an effect on the number of story elements and the storytelling characteristics (RQ2).

## Methods and materials

### Study design

An online survey was used to investigate the effect VN on small stories of older adults by collecting their responses after being exposed to a VN stimulus. When viewing the VN stimulus (a video), participants were instructed: ‘Image yourself sitting on this bench with someone else. What would you talk about?’, after which participants typed in their response. To investigate the effect of the VN characteristics mystery and spaciousness a between-subjects design was used. Participants were randomly assigned to one of four conditions in a 2 (mystery: low vs. high) × 2 (spaciousness: low vs. high) design. For further details, please see the Stimuli, Outcome measures and Procedure sections.

### Stimuli

**Figure 1.**
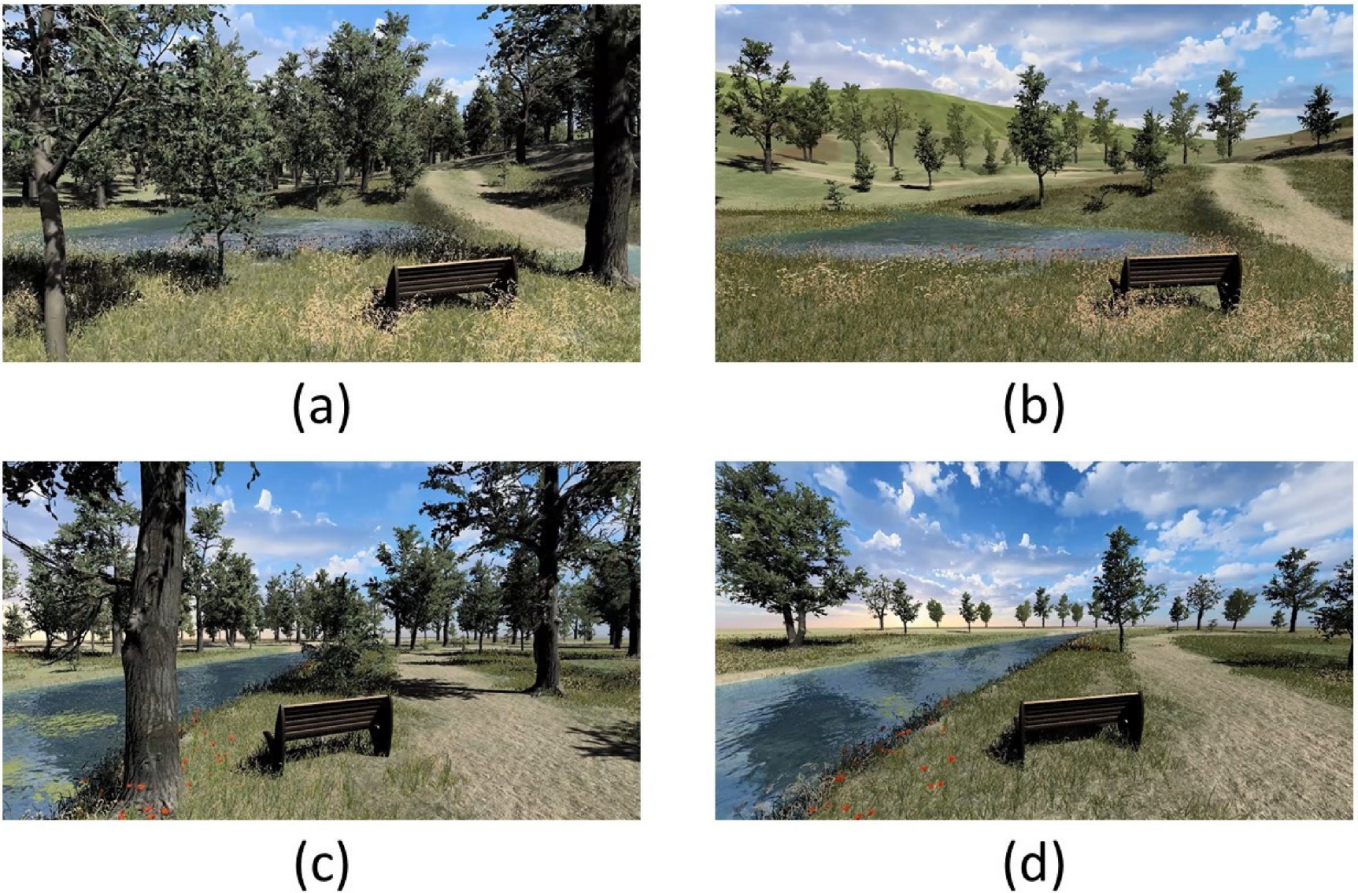
Screenshots of the VN videos.

*Note.* Four VN videos were created based on the variable’s mystery: high (a, b: presence of hills) versus low (c, d: absence of hills) and spaciousness: high (b, d: low tree density) versus low (a, c: high tree density). © The Author(s) 2024.

VN videos were created with purpose-built software using Unity3D (https://unity.com) at the University of Twente (https://bmslab.utwente.nl/virtual-nature-healing-environment/). This software can create VN environments and export them as videos and images. *Mystery* was manipulated with the presence or absence of hills, as hills obscured parts of the horizon from view, in line with previous research that also used nature characteristics to obscure the view.^37^ *Spaciousness* was manipulated with low and high tree density, resulting in an open or more dense scene.^54,55^ In the VN videos these two characteristics could be present or not, thus leading to four designs. The VN videos were animated; clouds were rolling by and leaves, flowers and grasses were moving to suggest the presence of wind. In addition, from a royalty-free database (https://www.soundfishing.eu/) an audio file with calm wind and bird sounds was edited into all VN videos to create a more realistic and immersive experience, as multimodal stimuli generate higher levels of immersion.^56^ The duration of the VN videos was 25 seconds. Figure 1 displays screenshots of the VN videos.

### Outcome measures

#### Story elements

The story elements were measured using the presence (or absence) of four basic elements of narrative: ‘situatedness’, ‘event sequencing’, ‘worldmaking/disruption’ and ‘what it's like’.^47^ For each response, a sum score was calculated ranging from 0 to 4; a higher sum score means that the response is more in line with a prototypical story structure.

#### Storytelling characteristics

Storytelling characteristics were measured using three variables: ease of storytelling, valence and social intention. ‘Ease of storytelling’ was measured with the statement: ‘I found it very easy to write down this story’ on a 5-point scale: (1) fully disagree – (5) fully agree.

‘Valence’ of the story was measured with an adjusted Dutch version of the Positive and Negative Affect Schedule (PANAS),^57^ using five positive (pride, cheerful, enthusiasm, inspired, determined) and five negative words (nervous, upset, fear, shame, restless). With the statement ‘Below are ten words that represent feelings or emotions. Report how well each word fits with the story you just wrote down’ on a 5-point scale: (1) not at all/hardly, (2) a little, (3) average, (4) good, (5) very good. For both the positive and negative words, a sum score ranging from 5 to 25 was calculated and used for analyses. A higher score means a more positive or more negative valence. Both positive and negative valence were found reliable (Cronbach's alpha of the current sample, respectively: 0.80 and 0.77).

‘Social intention’ of the story was measured with five statements: ‘I would like to share this story with someone else, like family or friends’, ‘I would use the story I just wrote down to … develop more trust in a relationship, … have closer contact with someone else, … share memories to foster friendships and … invite someone to tell more about themselves’. Participants responded to the statements using a 5-point scale: (1) fully disagree – (5) fully agree. A higher score means a person has more intention to use the story for social interaction. Principal component analysis revealed one component (eigenvalue > 1.0) explaining 64% of the variance, which was found reliable in the current sample (Cronbach's α = 0.85).

#### Nature-relatedness

Nature-relatedness reflects an individual's connectedness with the natural world^42^ and was measured using the short version of the Nature Relatedness Scale,^58^ consisting of six items such as ‘My relationship with nature is an important part of who I am’ and ‘My ideal vacation would be a remote, wilderness area’. A higher score means that a person feels more connected to nature (Cronbach's α current sample = 0.79).

#### Available nature opportunities

To control for available nature opportunities, a multiple-choice question was used: ‘Which nature opportunities are currently available to you?’ with six options: private balcony, private garden, communal garden, nearby park, nearby nature area and other. Participants could select more than one option leading to a sum score ranging from 0 to 6 for each response; a higher score means that a person has more nature opportunities available.

### Participants

Due to the explorative character and online design of the current study, recruitment was executed via an independent non-profit organization in The Netherlands by sending email invitations to eligible participants (aged 60 years and over) in their research panel. No further inclusion or exclusion criteria were applied. In total, 323 responses were collected of which 167 were incomplete. Another 23 responses had to be excluded due to using a mobile device (e.g. smart phone or tablet). Additionally, ten participants did not give consent and five participants were excluded because they did not see the video properly. Therefore, the final analyses included 118 participants. Measured demographic characteristics were age, sex, highest educational degree, household (single or multiple persons in their household) and residential situation (independent, partially dependent or fully dependent). Table 1 shows the demographic characteristics of the included participants and corresponding statistics. Randomization did not lead to significant differences between the four experimental conditions (*p* > 0.05) on demographic characteristics, nature-relatedness and available nature opportunities.

**Table 1.** Overview of the demographic characteristics of the participants.

Demographic characteristic	Specification		Comparison^a^	
			Test statistic	*p*-Value
Age	Years, mean (*SD*)	76.8 (4.5)	*F*(3, 114) = 0.543	0.654
Sex	Female (%)	27	χ^2^(3) = 4.265	0.234
	Male (%)	73		
Educational degree	Primary or secondary school (%)	17	χ^2^(6) = 7.014	0.320
	Vocational degree (%)	25		
	College or university degree (%)	58		
Household	Single person (%)	27	χ^2^(3) = 2.296	0.513
	Multiple persons (%)	73		
Residential situation	Independent (%)	100	n.a.	n.a.
Nature-relatedness	Mean (*SD*)	3.6 (0.6)	*F*(3, 114) = 1.609	0.191
Available nature opportunities	Mean (*SD*)	2.0 (0.9)	χ^2^(9) = 14.583	0.103

*Note*. Total number of participants is 118. VN: virtual representations of nature; n.a.: not applicable, residential situation did not vary between the four VN videos.

^a^ A comparison between all four VN videos on demographic characteristics was performed with analysis of variance (*F*) for age and nature-relatedness and Pearson's chi-square (χ^2^) test for sex, educational degree, household and available nature opportunities.

### Procedure

Eligible participants received an email with a link to the online survey. When entering the survey, participants were first briefed about the topic and aim. Subsequently, the survey software registered which device was used. Only desktop computers (PC) and laptops were allowed and mobile devices (e.g. smart phones or tablets) were rejected because these devices could not show the stimuli as intended. When participants used a mobile device, that response was registered and they received a notification explaining why the survey ended in addition to a link to start a new response on a PC or laptop. After briefing and the device check, informed consent was obtained. When consent was not given, participants received a notification why the survey ended.

When consent was given, participants were randomly assigned to one of the four experimental conditions. When viewing the VN video, the participant received an auditory instruction: ‘Image yourself sitting on this bench with someone else. What would you talk about?’ Subsequently, participants could type in their small story without a time or character limit. After the video, participants were asked whether they had seen the video properly and were able to type in their story. Secondly, the storytelling characteristics, demographics, nature-relatedness and available nature opportunities were obtained. Finally, after completion, participants were debriefed about the purpose of the study and were thanked for their contribution.

### Analyses

Randomization was checked by comparing the experimental conditions on demographics, ‘nature relatedness’ and ‘available nature opportunities’ using analysis of variance for age and ‘nature relatedness’ and Pearson's chi-square test for sex, educational degree, household and available nature opportunities.

To obtain information about the presence of the four story elements, the content of the small stories were coded with a deductive approach.^59^ Unit of analysis was the participant's response. The descriptions of the basic elements of narrative from were used as the coding frame to ensure structure and relevance.^47^ Thereafter the obtained story elements were subjected to content analysis using an inductive approach.^59,60^ The inductive approach also used the participant's response as unit of analysis and used three cycles to obtain relevant codes that represented the topic of each story element. To show what a story looks like when it is elicited by VN, the frequencies of the topic codes and four exemplary small stories (combining four, three, two or one story element, respectively) will be reported.

To test hypothesis H1 whether ‘nature relatedness’ has a predicting role when considering the number of story elements and the storytelling characteristics, first Spearman's rho (ρ) correlations were calculated. Secondly, a mediation analysis^61^ was performed to test whether ‘nature relatedness’ has a direct or indirect effect. In the mediation analysis (model: 4; bias-corrected 95% confidence intervals (CI); 5000 bootstrap samples) ‘nature relatedness’ was the independent variable as it could be considered a demographic factor. ‘Social intention’ was considered the dependent variable as this storytelling characteristic is most in line with the current study's aim: intention to use the story for social interaction, i.e. eliciting storytelling. ‘Sum of story elements’, ‘ease of storytelling’, ‘positive valence’ and ‘negative valence’ were considered mediators as these variables can be considered properties of the small stories.

To test H2 and RQ2 concerning the effects of ‘mystery’ and ‘spaciousness’, an analysis of covariance was used with the independent variables ‘mystery’ and ‘spaciousness’ and the dependent variables ‘sum of story elements’, ‘ease of storytelling’, ‘positive valence’, ‘negative valence’ and ‘social intention’. Covariates were ‘nature relatedness’ and ‘available nature opportunities’.

Statistical analyses were performed with IBM SPSS Statistics version 28. For all analyses, the significance level was set at *p* < 0.05. In addition, for the mediation analyses effects were also found significant if the upper and lower limit of the CI did not include the value zero, i.e. ruling out zero as a possible value of the effect.^61^

## Results

### Story elements

Table 2 shows the mean and percentages of the sum of story elements and the presence of each individual story element. The deductive analysis showed that three (3%) responses did not have any story elements, 12 (10%) had one element, 50 (42%) had two elements, 38 (32%) had three elements and 15 (13%) had all four elements. Of the three responses that did not contain any story elements, one stated that it would depend on the person to talk to and the other two could not think of a story to share with someone. Therefore, the research question (RQ1) whether VN are able to elicit small stories can be affirmed since most (97%, *N* = 115) of the responses contained at least one story element.

**Table 2.** Descriptive results of story elements and storytelling characteristics.

			Total	Mystery		Spaciousness	
				High	Low	High	Low
			*N* = 118	*N* = 61	*N* = 57	*N* = 57	*N* = 61
Story elements (%)	Sum (%)	0	3	5	0	0	5
		1	10	8	12	7	13
		2	42	41	44	46	39
		3	32	36	28	33	31
		4	13	10	16	14	12
	Situatedness (%)		89	92	86	97	82
	Event sequence (%)		27	31	23	30	25
	Worldmaking/disruption (%)		48	41	56	46	51
	What it's like (%)		79	75	83	83	75
Storytelling characteristics (*M*, *SD*)	Ease of storytelling		3.6 (1.1)	3.6 (1.2)	3.7 (1.0)	3.6 (1.1)	3.7 (1.2)
	Positive valence		13.0 (4.7)	13.3 (4.9)	12.8 (4.5)	13.2 (4.3)	12.9 (5.0)
	Negative valence		6.7 (2.8)	6.3 (2.6)	7.0 (3.0)	6.5 (2.4)	6.8 (3.1)
	Social intention		3.0 (1.0)	2.9 (1.0)	3.0 (1.0)	2.9 (0.9)	3.0 (1.1)

*Note*. Sum (%) shows the percentage of the small stories containing respectively 0, 1, 2, 3 or 4 story elements.

In Table 3, the topics per story element and their frequencies are shown. The small stories were predominantly situated in the presented VN video itself, as indicated by direct references to the VN (*N* = 77), e.g. by using words like *here*, *there* or *this landscape*. Some participants mentioned specific elements of the VN, such as the hills and the nature sounds. For instance, one participant mentioned:

Good afternoon. Also going for a walk? Beautiful spot here with that little fen. It's just a pity that the view is limited by the hills around. This way you have no idea whether you are situated low or high. Of course we came here climbing or just walking, but you lose that feeling now when you sit here. (Male, 79 years)

**Table 3.** Frequencies of mentioned topics per story element.

Story element Situatedness	*N*	Event sequence	*N*	Worldmaking/disruption	*N*	What it's like	*N*
In video itself	77	Memory/the past	10	Meaning making	12	Calmness	45
In nature	20	Present and the past	7	Concerned about nature's future	11	Happiness	30
During a walk	2	Present and the future	5	Social contact	7	Beauty	30
In the Netherlands	1	Based on the environment	2	Mysterious landscape	6	Fear	4
In an environment	1	Sitting on the bench	2	Feeling rushed/busy cities	4	Lack of nature experiences	4
Overlooking the water	1	Present conversation	2	Corona-pandemic	4	Solitude	3
Corona-pandemic	1	A walk similar to others	1	This is not real nature/real nature is best	4	Missing something	3
Would rather not talk	1	Past and future	1	Everyday quarrels	2	Boredom	3
		Newly occurring thoughts	1	Health	2	Frustration	2
		Past, present and future	1	Nature triggering memories	2	Holiday	2
				Positive/peaceful nature	2	Distraction by nature	1
				Quietness/no talking	2	Naturalness	1
				Availability of nature	1	Downplaying	1
				Politics	1	Anger	1
				Virtual nature for city residents	1	Joy	1
				Walking, not sitting on a bench	1	Sorrow	1
				Concerned about grandchildren's future	1	Freedom/spaciousness	1
						Regret	1
						Amazement	1
						Warm feelings	1

Moreover, the events were mostly sequenced using memories (*N* = 10). Furthermore, the small stories were used most for meaning making in life (*N* = 11) and to communicate worries about the future of nature (*N* = 10). Finally, feelings of calm (*N* = 45), happiness (*N* = 30) and beauty (*N* = 30) were used most often to express the storyteller's experience. Especially emotional words such as *soothing*, *peaceful*, *enjoying*, *nice* and *beautiful* were used to describe the appearance of the VN, e.g. a participant mentioned:

I am sitting on the bench with a sweet, nice woman and ask her if she would like to go on holiday to [Dutch province] with me. The tranquillity that radiates from the area surrounding the bench gives me that idea. We haven't known each other that long, but I think we both will have a nice holiday in [Dutch province]. (Male, 71 years)

For exemplary purposes, four small stories are quoted: one combines all four story elements and the other three combine either three, two or one story element, respectively. The story elements are highlighted in brackets. Moreover, in Appendix 1, all collected small stories containing all four basic elements of narrative (*N* = 15) are shown.

Small story with all four elements:

What a lovely landscape, nowhere a house or traffic [situatedness]. What a tranquil place [what it's like]. No stripes of airplanes. I would like to take a swim in the water. I used to cycle 12 km from school to home, through the meadows. When the weather was nice, halfway, I just laid down in the grass in the sun and started thinking, next to my bicycle (I poetically called it a ‘dune slack’). When I am here with you, I start thinking about the tranquillity of then [event sequencing]. We nowadays hardly have the time to quietly lay on our backs and look at the clouds. I get a feeling of longing to those days. At the same time, a feeling of worry starts, that this might disappear [what it's like]. That our children will have to miss this [worldmaking/disruption]. Yet, I mostly get a feeling of inner peace, peace of mind [what it's like]. It strengthens my thought that briskly walking in nature wills solve most problems. It puts things in perspective [worldmaking/disruption]. I then also think of my father, who was a biologist or better put: a super nature person. He gave during walks so much information on trees and plants, that it went in one ear and out the other [event sequencing]. I regret that now. Now I cannot name even the simplest of trees for my (grand)children. I wish I could ask him! [what it's like]. (Male, 74 years)

Small story with three different story elements:

About nature in the first place. How beautiful and calming this can be, just look at all the different colours. Do you see the red cornflowers? On the water's edge? And how the blue sky is being mirrored by the water [situatedness]? It exudes calmness, but also a bit abandonment [what it's like]. Does it wait for something? A good place to think about anything. But also with someone on the bench to talk about inner peace compared to everyday ‘hectic’ life. How many people on this earth miss this peaceful environment? The why. Should you care? Are you not someone who is lucky? To sit here with someone and to able to talk freely about anything [worldmaking/disruption]. (Male, 83 years)

Small story with two different story elements:

What a lovely place [situatedness], do you also hear the birds whistling? It looks like a foreign country. I feel like I am on holiday [what it's like], what about you? (Female, 74 years)

Small story with one story element:

The water is flowing and the trees are whistling. The wind also blows a fair bit. [situatedness]. (Male, 75 years)

### Storytelling characteristics

Concerning ‘ease of storytelling’, on average participants scored 3.6 (*SD* = 1.1) on a scale of 1–5. Looking at response options showed that most participants (67%) fully agreed or agreed it was easy to write down the story, 17% were neutral and 16% disagreed or fully disagreed. Concerning ‘valence’, on average participants rated their stories 13.0 (*SD* = 4.7) for positive valence and 6.7 (*SD* = 2.8) for negative valence on a scale of 5–25. This difference was statistically significant: *t*(1117) = 13.573, *p* < 0.001, meaning that the reported small stories of the participants were predominantly positive in character. Concerning ‘social intention’, participants scored on average 3.0 (*SD* = 1.0) on a scale of 1–5, showing that participants had no strong intention to either use or not use this story for social interaction.

### Nature-relatedness

For hypothesis H1, first Spearman's ρ correlations were explored (Table 4). Participants who felt more related to nature, had more story elements in their story (ρ = 0.203, *p* = 0.028), gave their story a higher positive valence score (ρ = 0.210, *p* = 0.022) and had more intention to use their story for social interaction (ρ = 0.256, *p* = 0.005).

**Table 4.** Exploratory Spearman's rho correlations.

	2	3	4	5	6
1. Nature-relatedness	0.203*	0.062	0.210*	0.056	0.256**
2. Sum of story elements	–	0.195*	0.046	0.115	0.189*
3. Ease of storytelling		–	0.094	−0.143	0.202*
4. Positive valence			–	0.181*	0.414***
5. Negative valence				–	0.177
6. Social Intention					–

* *p* < 0.05; ** *p* < 0.01; *** *p* < 0.001.

In addition, participants with more intention to use their story for social interaction, also have more story elements in their story (ρ = 0.189, *p* = 0.040), found it easier to write down their story (ρ = 0.202, *p* = 0.028) and gave their story a higher positive valence score (ρ = 0.414, *p* < 0.001).

**Figure 2.**
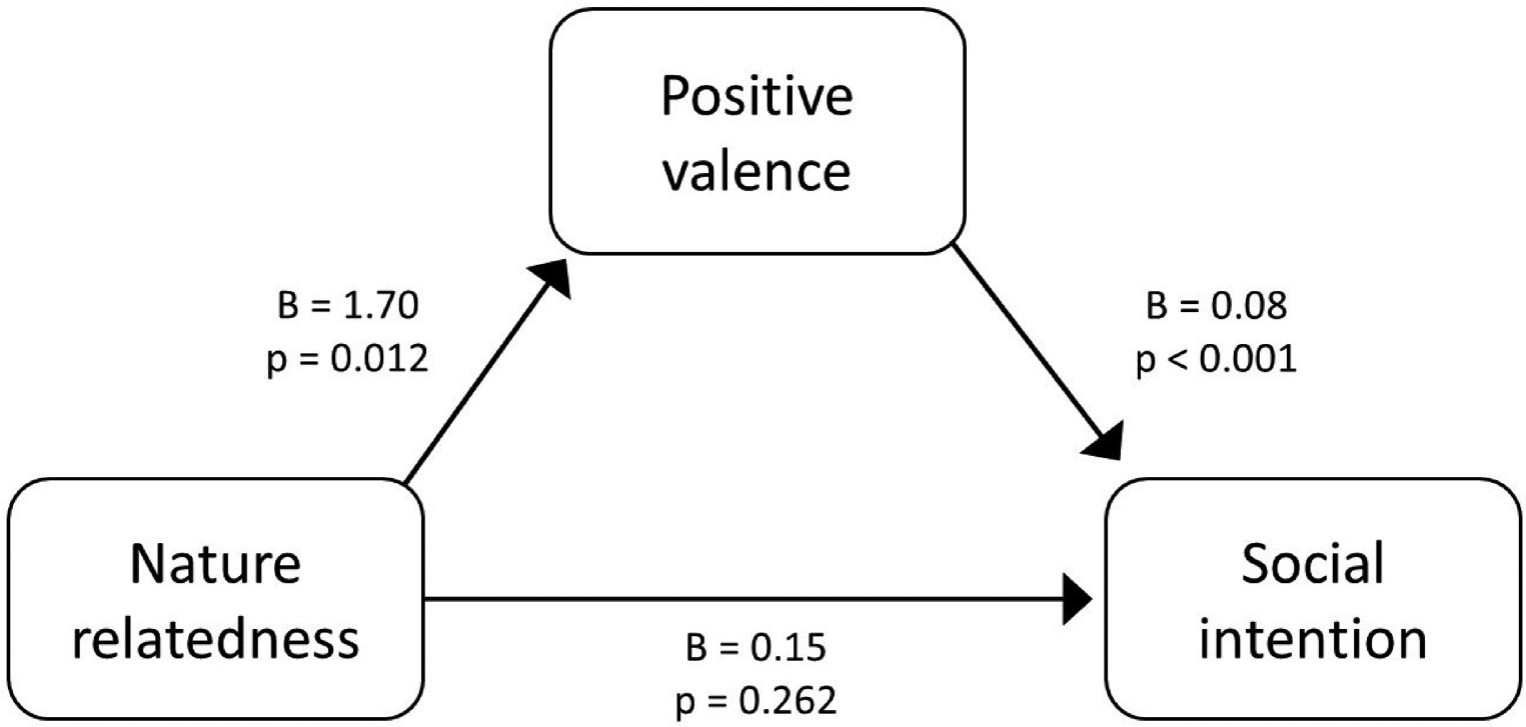
Effect of nature relatedness on social intention mediated by positive valence.

*Note.* Unstandardized coefficients with significance values are given.

The mediation model showed that ‘nature relatedness’ did not have a direct effect on ‘social intention’: *B* = 0.15, SE = 0.13, CI = −0.11 to 0.41, *t*(112) = 1.13, *p* = 0.26). ‘Nature relatedness’ did have a significant effect on ‘social intention’ when mediated via ‘positive valence’: *B* = 0.13, SE = 0.06, CI = 0.02–0.27. This full-mediation model (Figure 2) significantly explained 27% of the variance, *F*(5, 112) = 8.108, *p* < 0.001: when participants feel more related to nature, they also assigned more positive valence to their story and thereby, had a higher intention to use their story for social interaction.

### Mystery and spaciousness

No significant main effects of both mystery and spaciousness and no significant interaction effects were found on the sum story elements and storytelling characteristics (Table 5), meaning that H2 and RQ2 were both rejected.

**Table 5.** Statistics of main and interaction effects of mystery and spaciousness.

		Mystery			Spaciousness			Interaction effect		
		*F*	*p*	*η* ^2^	*F*	*p*	*η* ^2^	*F*	*p*	*η* ^2^
Story elements	Sum	0.407	0.525	0.004	3.668	0.058	0.032	0.415	0.521	0.004
Storytelling characteristics	Ease of storytelling	0.068	0.795	0.001	0.354	0.553	0.003	0.397	0.530	0.004
	Positive valence	0.667	0.416	0.006	0.405	0.526	0.004	1.614	0.207	0.014
	Negative valence	1.294	0.258	0.011	0.060	0.808	0.001	1.849	0.177	0.016
	Social intention	0.020	0.888	<0.001	0.045	0.833	<0.001	0.020	0.887	<0.001

*Note*. Degrees of freedom for all *F*-values: 1, 116.

## Discussion

The aim of the current study was to explore VN, implemented as computer-animated nature videos, as a means to elicit storytelling in older adults. Although only few participants wrote down a small story containing all four elements, in the vast majority of participants the used VN were able to elicit small stories containing at least one basic story element. Moreover, VN was a fruitful source for storytelling because the majority of the participants found it easy to come up with and write down a story, situated their story in the presented VN and wrote stories with an overall positive character. In addition, it seemed that not so much the characteristics of nature (mystery and spaciousness) as the characteristics of the participants (nature-relatedness) played an important role in eliciting and sharing small stories.

In the current sample, coming up with and writing down a story was considered easy and almost all participants incorporated at least one basic element of narrative. Although this result cannot easily be related to previous studies on storytelling, natural environments can provoke highly reflective thoughts with respect to, e.g. memories, spirituality, life and the world.^7,40^ This underscores that evoking a small story using VN is feasible. Moreover, considering storytelling characteristics, the currently used VN were able to elicit small stories and they contained significantly more positive than negative valence, in accordance with previous research.^7,39^ This can partly be explained by similar methodology,^39^ but also by the Biophilia theory^62^ and ART,^34^ which share the basic understanding that humans ‘love’ nature environments and automatically associate them with, e.g. prospect or safety. In addition, positive phrasing in storytelling is related to positive health outcomes,^63^ while reminiscing about negative events can have adverse effects on mental health.^64^ Importantly, current results showed that more positive valenced stories were more likely shared with others. In sum, it strengthens the existing notion that VN can be used to increase social well-being,^25^ by eliciting and sharing positively valenced associations and small stories, and that VN-based storytelling could be used alongside real nature-based interventions, such as horticulture therapy or nature-based social prescribing.^14,65^

The current findings further indicate that nature-relatedness is an important condition for nature-based storytelling to occur; people who feel more related to nature have more basic story elements in their story, more positively valenced stories and more intention to use their story for social interaction. This is in line with previous research revealing the effect of nature-relatedness on our behaviour^33,58^ and associations with VN.^39^ These findings indicate that for VN to have a beneficial effect on social interaction and social connectedness, it is important that a person feels related to nature. Therefore, although according to the Biophilia theory^62^ humans have innate affiliations with nature environments, they do not have the same effect in all of us making it paramount to acknowledge that for nature interventions there is not a one-size-fits-all solution, as corroborated by previous literature.^29^ Interestingly, repeated exposure to VN increased nature connectedness in individuals with a low connection to nature,^66^ and even single exposure to VN showed this effect.^67^ This suggests that VN are a suitable method to increase social interaction by triggering storytelling, not just for individuals who feel related to nature as shown in the current study, but possibly also for those who do not (yet) feel related to nature. As such, exposure to VN is complementary to experiencing real nature environments: both can increase our connection with nature, and subsequently benefit our (social) well-being.^33^

Regarding the nature characteristics mystery and spaciousness no significant effects were found. This is not in line with previous research,^29,36–38^ but also incongruent with findings from a methodologically similar study.^39^ Arguably, the effects of these characteristics can vary with underlying concerns. For older adults with mobility constraints, safety concerns may be very prominent^17,18^ and translate to a preference for less mysterious and more secluded settings.^41^ For older adults with no such constraints, safety concerns may be less prominent and transpire in a preference for more mysterious and spacious settings. Possibly, our sample of older adults was heterogeneous with respect to their level of mobility, which might explain why no results were found for these nature characteristics. Clearly, these speculations warrant follow-up studies that take (potential) mobility issues into account. Another reason might be that the manipulation of the nature characteristics mystery and spaciousness was not profound enough. The exposure to VN via laptops and desktops, rather than large-scale screen projections, might have caused a rather low immersive experience.^56^

Regarding the story elements, it can be seen that the elements ‘situatedness’ and ‘what it's like’ were most frequently present in the small stories. For ‘situatedness’ this is likely due to the instruction (‘imagine yourself sitting on this bench with someone’), although participants were not required to actually mention this. The reason that the element ‘what it's like’ is frequently mentioned could be because of the positive valence of the small stories. The VN significantly triggered more positive valence than negative valence, corresponding to previous research,^39^ and is also reflected in the high frequency of the codes ‘happiness’ and ‘beauty’. Particularly, ‘beauty’ was often mentioned in relation to the nature scenery of the VN. Thereby linking the element ‘what it's like’ to the element ‘situatedness’ and explaining why both elements were often present in the small stories. An explanation for the lower presence of the story elements ‘event sequence’ and ‘worldmaking/disruption’ could be that people need time or practice to communicate a narrative. As narratives are usually obtained via several sessions of interviews and hence, are constructed over time by the storyteller.^46^

### Limitations

In addition to the aforementioned lack of immersion related to screen size, the lack of a control condition makes it hard to pinpoint whether the reported effects on storytelling are caused by the VN or by participating in the study. Arguably, a well-known city square might also provoke small stories useful for eliciting social interaction. However, our results show that nature-relatedness influenced storytelling and social intention, and that elements of the VN stimuli were incorporated into participants’ responses. These findings indicate that the nature context was critical. Future research should therefore continue to investigate nature environments to find out why and how they are beneficial for our social well-being.

Moreover, the participants could share their small story indirectly with the researchers, yet without any opportunity to interact due to the anonymous study design. While this setup is convenient for testing effects of VN characteristics in an efficient and ethical manner, it can be considered too far removed from having normal conversations in daily life, which may lower the current findings’ generalizability. Perhaps mentioning that the participants had to converse with an actual person, e.g. the researcher, could perhaps trigger participants (see the example with one story element) to incorporate more story elements because they were explicitly sharing their thoughts with someone.

### Recommendations for future research

Although the two VN characteristics did not result in differences with respect to the story elements used and the storytelling characteristics, other VN characteristics might also influence associations with nature. For example, more nature diversity in both flora and fauna is related to better psychological well-being;^68^ tended VN are related to more social aspirations when compared to wild VN;^54^ and the more beautiful nature is, the more prosocial behaviour is shown.^30^ Moreover, older adults may perceive established beneficial VN characteristics differently than younger adults, e.g. because of mobility issues.^18^ Therefore, the role of other VN characteristics than mystery and spaciousness should be established, how VN characteristics are perceived by older adults and to what extent differences between young and older adults can be traced to, among others, safety concerns.

Subsequently, as experimental exposure to VN can elicit small stories and in turn social intentions, a research design using VN in a more everyday setting will improve generalizability and establish the societal value of VN-elicited storytelling. Moreover, this would add to the existing body of research on social connectedness interventions that use nature environments by showing that VN is not only a complementary means to interact with and feel connected to nature, but also a way to make living spaces more suited for social interaction. For example, large-scale VN projections in the public areas of nursing homes might be a solution to counteract negative effects of feelings of loneliness among their residents.^69^

## Conclusion

This study showed that VN can elicit small stories in older adults. The majority of them found it easy to write down the stories and the small stories were mostly of positive valence. VN seem therefore a suitable method to promote storytelling. This effect was more pronounced in older adults who feel more related to nature, indicating the importance of nature-relatedness when one aims to use nature environments for social well-being purposes. Yet, whether the small stories elicited by VN also occur in face-to-face settings and promote social interaction requires future research.

## Appendix 1.

### Collected responses containing all four basic elements of narrative.

#### Small story 1

PP26; female, 72 years: I would talk about corona, that the virus is spreading again. And there are still people who do not see the usefulness of a vaccine. I am on a waiting list for an operation, everything takes longer before you have an appointment to see a doctor in the hospital, it takes almost 2 months. You have to wait longer for examinations in the hospital before you can go there and Corona is being blamed for everything. Now corona patients are coming into the hospital who did NOT want a vaccine but do burden other patients (who have been vaccinated) and now cannot be helped because the unvaccinated ones occupy the hospital beds/ICU. If you do not want to be vaccinated, then you must be brave enough not to be admitted, so that other patients do not have to wait longer.

#### Small story 2

PP30; male, 82 years: I would ask whether the person on the bench also has a problem with landscaped nature. Everything is too perfect to seem natural. Creation has nothing to do with order, straight lines and the human dimension. Anyway, I say – after his answer – the whole life is usually presented too well. That is also in our heads to make things more beautiful than reality. That is due to DNA and genes and our ranking in being on earth. Education also does not contribute to a vision of reality. But that beauty often does not match reality. We cannot make it more beautiful than reality. Reality, no matter how disorderly or chaotic it may appear, is always better than the polished image. Reality can only ask the right questions. To accept reality and feel liveable is actually the task of our stay on this planet. The bench we sit on and the ‘beautiful’ surroundings are actually fake. My neighbour on the bench says nothing to my story until the moment I say that this place has nothing to do with my reality. Constructed nature is not nature, I say a little softer than usual. Then she stood up and said: ‘Of course you're right’ and ‘that's why I'm going to walk a little further so that I can see the horizon better than here from the bench with a cackling man by my side.’ As she stood up and slowly walked away, I heard her say that I should really learn to think about life, nature and reality. Then a ‘goodbye and good luck with your own disorder. I'm going to try to think meaningfully about my future’, there behind those trees, where the horizon gives us light in the darkness.

#### Small story 3

PP33; male, 74 years: How beautiful and peaceful it is here. Listen to those birds. This is truly a place to relax and reflect on the difficult times we have left behind us and to think about the future. There should be much more of this nature so that our children and grandchildren can also enjoy it.

#### Small story 4

PP40; female, 72 years: How wonderful, such a moment of peace in these hectic times… I need that, but you know, right now I'm getting busier with all those online things, it's sometimes too much for me! The images do me good, as do the sounds of nature, but I don't have the peace yet (through the screen in this way) to really enjoy it… It just takes a lot of effort for me to really relax because there are so many things that require my attention, especially my mind, because I relax more with people, like now outside here. Then I think of those wonderful and relaxing walking holidays, where you can just do nothing in a place like this, muse, forget the time, in this togetherness with others, in peace and harmony.

#### Small story 5

PP49; female, 78 years: We talk about the tranquillity that the landscape radiates. It is beautiful, mild weather. We have time and everything invites us to talk about beautiful past times and precious memories. That brings up warm feelings. It brings us closer together.

#### Small story 6

PP51; male, 77 years: Sitting in nature gives me peace and a feeling of satisfaction with my life. In addition, my youth came back, my first girlfriend on a bench (at that time that was a bit secretive)

#### Small story 7

PP55; male, 87 years: It's nice to meet someone. Here I find peace to think about the future. How can I best photograph this environment to bring back the memory of it. I also remember the story that was told here.

#### Small story 8

PP64; male, 89 years: What a peaceful place to muse about how our lives are going. How good we had it in the past but also what sad moments we had with and about our children. How difficult it is right now, now that you are in a nursing home and we can no longer live together.

#### Small story 9

PP65; male, 72 years: What a lovely landscape and not a house or traffic to be seen anywhere. What peace. No airplane streaks. I would like to swim in the water for a while. I used to have to cycle 12 km from school to home, through meadows. When the weather was nice, I would just lay down halfway in the grass in the sun and think, next to my bicycle (I poetically called it a ‘dune pan’). When I sit here with you, I am reminded of that peace of that time. Now we hardly get around to quietly looking at the clouds on our backs. I'm getting kind of homesick for the past! At the same time, I worry that all this might disappear. That our children may have to miss this. Yet above all, I get a feeling of inner peace, peace of mind. It reinforces my belief that taking a long walk in nature solves most problems. It puts things into perspective. I then also always think of my father, who was a biologist or rather a super nature person. During walks, he gave so much information about trees and plants that it went in one ear and out the other. I regret that now. I cannot tell my own (grand)children the name of the simplest tree. If only I could ask him!

#### Small story 10

PP76; male, 75 years: That of course depends on who is sitting next to you. But soon, it's about ‘wonderful, what peace and quiet here. nice spot here, right on the water, no noise, you hear the birds chirping. Wonderfully relaxing and wonderfully unwinding. I think this will bring back feelings of similar moments in my life. Especially a feeling of relaxation and enjoying the pristine. can also be a setting for discussing some problems in a relaxed manner. Without being distracted or influenced by the issues of the day. And also to realize with how little (in a material sense) you can feel happy and enjoy.

#### Small story 11

PP77; male, 75 years: First of all, I would consider ourselves lucky to have friendly weather. We can consider ourselves lucky that we can enjoy the peace and quiet. Not only here in the great outdoors, but also that we can spend our free time as we wish. However, we still have to take the Covid-19 pandemic and the restrictions that come with it into account. It is desirable that as many people as possible want to be vaccinated, so that the remaining measures can also disappear. I'm worried about the situation in the world. When I think about the conflicts and wars that are still going on and the hardening of leaders’ positions against each other, I feel gloomy. The fact that far too little is being done to prevent natural disasters also makes me anxious. Our grandchildren face an uncertain future. As older adults, we are partly to blame for having plundered nature and its resources. The interests of trade and industry have been placed by politicians above those of the general well-being. That is also the reason why we no longer trust politics and politicians. The power belongs to money and no longer to the well-being of people.

#### Small story 12

PP85; female, 83 years: If I am sitting on the bench with someone, I would talk about what the person is telling me, for example, which will usually be a personal story about something that just happened or what is on someone's mind. But if I see a bird flying, I will be distracted and immediately point it out, after which we continue with the story. When the conversation on the subject has been completed, I have probably already seen many other things, e.g. a grass or flowers or insects that we can then talk about for a while and which then give rise to a story from the past or something that just happened can be stirred up and talked about. Memories are associated with many visible things.

#### Small story 13

PP89; female, 82 years: Nice question! Depends a lot on who I'm sitting on the bench with. So I now have to make a choice with whom I am/will be talking. I choose a neutral conversation with a man my own age with whom I walk and photograph once a week. We often talk about politics, corona and what we came across in the newspaper last week. Less about our emotions, although we can occasionally react angrily when we talk about people who do not get vaccinated. We understand both sides. But we still hope that more people will have this done due to more admissions to hospitals and ICUs.

#### Small story 14

PP103; female, 73 years: About how the view is so beautiful and relaxing and that I miss that because we have been living in an apartment in the city centre for 6 years where the traffic drives you crazy (112 buses every day and then the other traffic and the noise of the people who walk past, also in the evening and at night, your sleep rhythm is disturbed because you keep waking up, I would give anything to reverse it, but that is no longer possible, the house prices have risen so fast that it is unaffordable for pensioners because we do not get a cent extra).

#### Small story 15

PP110; male, 73 years: I sit down on the right side of the bench and examine the painting in front of me. Why am I alone again, I can't share anything like this. The tranquillity and the view enhance the feeling of loneliness. I move to the middle, because there are no armrests anyway. It is beautifully maintained. I'm going home….
